# Controller Cyber-Attack Detection and Isolation †

**DOI:** 10.3390/s23052778

**Published:** 2023-03-03

**Authors:** Anna Sztyber-Betley, Michał Syfert, Jan Maciej Kościelny, Zuzanna Górecka

**Affiliations:** Faculty of Mechatronics, Warsaw University of Technology, 00-661 Warsaw, Poland

**Keywords:** cybersecurity, cyber-attack, fault detection, fault isolation, control loop performance, neural networks, linear models

## Abstract

This article deals with the cyber security of industrial control systems. Methods for detecting and isolating process faults and cyber-attacks, consisting of elementary actions named “cybernetic faults” that penetrate the control system and destructively affect its operation, are analysed. FDI fault detection and isolation methods and the assessment of control loop performance methods developed in the automation community are used to diagnose these anomalies. An integration of both approaches is proposed, which consists of checking the correct functioning of the control algorithm based on its model and tracking changes in the values of selected control loop performance indicators to supervise the control circuit. A binary diagnostic matrix was used to isolate anomalies. The presented approach requires only standard operating data (process variable (PV), setpoint (SP), and control signal (CV). The proposed concept was tested using the example of a control system for superheaters in a steam line of a power unit boiler. Cyber-attacks targeting other parts of the process were also included in the study to test the proposed approach’s applicability, effectiveness, and limitations and identify further research directions.

## 1. Introduction

Recently, apart from hazards related to equipment faults and human errors, there are also new threats [1,2,3] related to destructive targeted activities, such as cyber-attacks and sabotage actions. Both types of risks are particularly dangerous for critical infrastructures, such as the chemical industry, power plants, power grids, and water supply [1]. Despite various reasons, the effects of severe failures and attacks may be the same, e.g., fire, explosion, environmental contamination, destruction of the installation, and process stop.

In the FDI methods of fault detection and isolation [4,5], which have been developed for over 40 years, the analysed scope of diagnosis included process components, sensors, and actuators. Faults of these elements are a hazard to the proper functioning of the control systems. The correctness of the control algorithms in the FDI approach was not checked. On the other hand, the faults of the control units implementing the control algorithms were detected independently by the diagnostic software of these units using computer systems diagnostic methods. Diagnostics methods in the FDI community are still dynamically evolving, as indicated by review papers [6,7,8,9], and it is worth drawing inspiration from the community achievements.

Independent of the FDI methods, another research direction was developed, which can be described as the control loop performance assessment. In large-scale industrial processes, operators and control engineers have an increasing number of control circuits under supervision (from 30 to 2000, according to [10]). Therefore, automated methods for evaluating the performance of the loops are needed.

Many indicators have been developed in the literature to diagnose the most common problems in control circuits. Some of these indicators can also be a significant alarm symptom in the event of cyber-attacks.

The fundamental problem in assessing the operation of the controller is whether the poor quality of regulation is the result of internal problems or the influence of external disturbances. The initial work of [11] proposed comparing the operation of the regulator with the operation of the minimum variance control.

The control loop performance assessment system should be able to work on data from the system’s regular operation in a closed feedback loop. The currently existing solutions include tuning quality assessment, detecting and isolating faults, and searching for the source of plant-wide disturbances. The state-of-the-art has been described in review articles [10,12] and monographs [13,14].

The following practical problem is essential: how to increase the security of industrial control systems (ICS) in the case of threats related to faults of technical equipment, sensors, and actuators, human errors, and destructive targeted actions, such as cyber-attacks and sabotage actions. The current solutions are not satisfactory because they relate to specific threats. There are no holistic solutions that would comprehensively address security issues. One of the partial elements of this problem is finding effective methods of detecting intrusions into the control system, consisting of malicious modifications of the control algorithm or its parameters.

This paper is an extension of the conference paper [15]. The main contributions of this paper are as follows:

- The concept for the use of control quality indicators in cyber-attack detection, understood as the detection of the cyber-attack itself or the detection of its components (partial actions);
- a combination of control quality indicators with controller modelling;
- preprocessing and combining indicators to obtain interpretable diagnostic signals;
- experiments and analysis in distinguishability of the components (partial actions) of cyber-attacks;
- publication of a dataset with cyber-attack scenarios for the superheater system available at https://doi.org/10.5281/zenodo.7612269.

**Figure 1:**
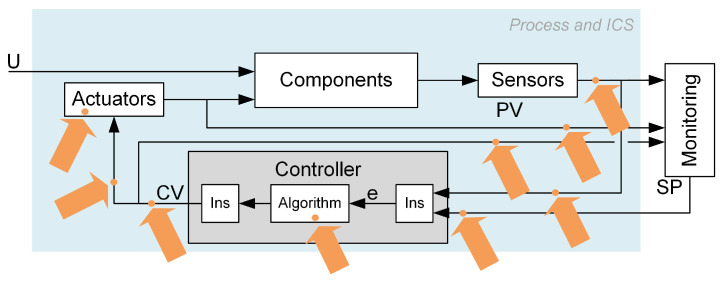
Cyber-attack detection and isolation scheme based on controller modelling and loop performance indices.

The main idea of cyber-attack detection based on controller modelling and loop performance indicators is illustrated in Figure 1.

**Figure 2:**
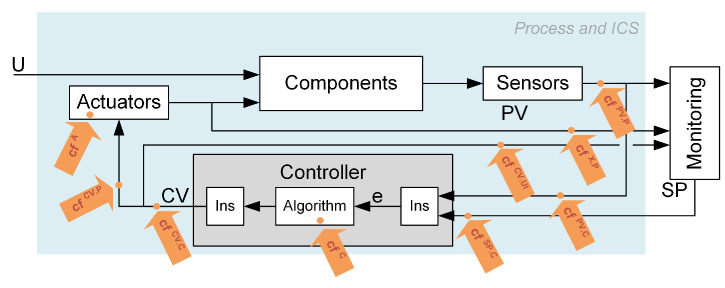
Symbolic places of injection of attacks.

Cyber-attacks entering the control system through the protection rings may destructively distort the measurement and control signals, the control algorithm, or its parameters. Potential attack vectors are illustrated in Figure 2. Each cyber-attack is carried out according to a designed scenario—a specific method of attack. Such a scenario consists of elementary impacts (partial actions) on individual system elements and signals in communication channels called cybernetic faults. “Cybernetic faults” plays an analogous role as “process faults” used by the FDI community. They represent a specific cause of the erroneous operation (or falsification of values) introduced by the attacker as a single step of the attack. The primary presented idea is to focus on the detection and isolation of cybernetic faults. However, the detection of any of the cybernetic faults equals the detection of a cyber-attack.

Therefore, real-time supervision of the control loops becomes necessary to detect intrusions into the control system early. The diagnostic system should detect and isolate both faults and attacks or its components.

This work aimed to develop and test algorithms to detect cyber-attacks and/or its components, i.e., cyber-faults, aimed at control algorithms. The paper shows that attack-detection methods based on controller models and control loop performance indicators are effective in this task. The attacks may, for example, modify the algorithm’s parameters. The change in the normal-reverse regulator operation mode leads to positive feedback. Changes in the settings may also result in the loss of system stability, and the modification of the setpoint value may result in the process entering the emergency area.

We also consider attacks directed at actuators, masking control values entering the process and PV or SP values entering the controller. We demonstrate effective detection in most cases and show further directions of research.

A case study was used as the research method. The possibilities of solving the formulated research problems were analysed on the example of a superheater system.

The proposed approach differs from the existing solutions in the following aspects:

- The approach is directed at the industrial controller.
- Only data from regular plant operations are needed.
- There are no specific requirements regarding the controlled process.

The structure of the paper is as follows: Section 2 presents the state-of-the-art in cyber-attack detection in control systems, Section 3.1 presents control loop performance indicators, and Section 3.2 introduces proposed controller modelling approaches. Preprocessing and merging of indicators are described in Section 3.3. Section 4 presents the case study, Section 5 the results, Section 6 shows a discussion of the results, and Section 7 concludes the paper.

## 2. Detection of Cyber-Attack on Control System

The protection of the control system against cyber-attacks is achieved primarily by using demilitarised zones, firewalls, data encryption, VPN (IPsec) networks, network segmentation, identity verification, access authorisation, and password management. Cyber-attack detection is carried out by monitoring network traffic that does not allow the detection of all anomalies [16]. The solutions used in IT systems do not guarantee the lack of possibility of an attack getting into the control system. Thus its destructive impact on the controlled process [17]. Early detection of anomalies and their isolation is essential to effectively respond to emerging hazards and threats.

The issues of cyber-attack detection in industrial control systems are developed very intensively [17,18,19,20,21]. Intrusion detection techniques against cyber-attacks are mainly divided into two categories: based on signatures and anomalies. Signature-based methods require a model of the system’s functioning in the case of a given type of attack. Anomaly-based attack detection techniques detect deviations from normal system behaviour. Similar approaches are used in fault diagnosis by the FDI community.

In terms of the detection of cyber-attacks in ICS, the following research directions can be distinguished: detection based on network traffic exploration, the analysis of network protocols, and the analysis of process data. ICS attacks often cause unusual network traffic or violate network protocol specifications. The methods of detecting these anomalies are derived from the methods used in IT systems. The above approaches are often used in Intrusion Detection Systems (IDS) [22]. Article [23] presents classifications of cyber attacks in network control systems and cyber-physical systems (CPS).

If the attacks penetrate the control system through the security measures applied, they affect the functioning of the control systems. Therefore, they can be detected as a discrepancy between the observed and reference activity, represented by models characterising the normal state of the controlled process. The detection scheme presented in [24,25] is identical to the fault detection scheme used for a long time in FDI methods.

There are passive and active approaches to detecting cyber-attacks. Passive methods are based on operating signals. Active methods require introducing an appropriate input signal and the analysis of its influence on the control systems. The active approach was presented in [26,27,28]. Observers [29,30], Kalman filters [31,32], and machine learning methods [33] were used as passive detection approaches. The use of neural models to detect anomalies was the subject of works [34,35]. Paper [36] investigates the attack isolation and attack location problems for a cyber-physical system based on the combination of the H-infinity observer and the zonotope theory. For anomaly detection, quantitative and qualitative models can be used. The works [2,37,38] present models in the form of rules that detect faults and attacks that cause the reverse controller operation or the actuator block. Developing benchmarks for testing and comparing cybersecurity solutions is also an active research area [39,40].

Research on cyber-attack detection tends to focus on a specific type of attack. The most significant amount of work is related to detecting false data injection attacks [34,41,42,43,44,45,46]. Fewer publications present studies of detection methods for replay attacks, covert attacks, and zero dynamics attacks [47].

Research on attack isolation is also emerging. The Anomaly Isolation Scheme (Iterative Observer Scheme) proposed in paper [48] is an extension of the well-known Generalised Observer Scheme, which was used to isolate single-sensor faults. The Unknown Input Observer is used to isolate cyber-attacks. In [49], a methodology based on the cyber-physical systems two side filters and an Unknown Input Observer-based detector has been proposed. A linear time-invariant (LTI) model was used, considering the impact of faults and cyber-attacks.

Our proposed approach does not require knowledge of process models with the impact of attacks and faults, nor does it assume a specific process form (such as LTI). All that is required is standard archived control loop data in the form of PV, SP, and CV signals and, for isolation, a binary diagnostic matrix, which is much simpler to obtain than the whole model.

Recently, attack detection based on control loop performance indices was proposed [50]. This method uses the Harris index, which, in its base version, was designed to fixe SP control systems. Additionally, the value of the Harris index is sensitive to the level of disturbances. Our goal is to propose a solution suitable for cascade control systems and includes isolation analysis. Therefore, we decide to rely on a larger number of simpler indices.

The detection methods derived from the approaches known in IT networks have already reached a high level of advancement, measured by many publications and the offered IDS systems using these techniques. The solutions derived from the approaches developed in the automation community, including intrusion detection based on process data analysis and control loop supervision, are more recent and less documented in publications. In the opinion of the authors of this paper, these methods that control the integrity of process data collected from measuring devices, actuators, and controllers may be of great practical importance. Methods developed based on fault diagnostics and models linking process variables can be used for this task.

The introduction of diagnostic systems that recognise both faults and cyber-attacks will constitute an additional layer of operational security (Layer of Protection) and another Ring of Protection in ICS.

## 3. Methods

### 3.1. Loop Performance Indicators

Many different indices of control quality are used in the literature and industrial software. Table 1 presents a list of indices, by category, that have been selected for initial testing. The indicators used in the final solution are indicated by ${}^{*}$. Indices in use are described in this section. For information regarding other indices, the reader is referred to the cited papers. During the preliminary tests, we found that the more complex indices (Harris index, oscillation factors) perform adequately, but their values are more challenging to interpret. In the application under consideration, we are mainly interested in the change in control quality and system behaviour. For this purpose, using more straightforward statistics for the control signal and control error is sufficient. These indicators are easy to interpret and calculate and do not require parameter selection. In addition, a set of dedicated indicators was also determined from the controller models.

Indicators determined based on the response to a step change in SP were excluded due to the difficulty of application in the presence of significant disturbances and the inapplicability in fixed setpoint control systems or for auxiliary controllers in cascade control.

In the group of dedicated indicators, the estimation of the derivative time $T_{d}$ was omitted due to numerical difficulties when pre-filtering the signals fed to the controller.

In Table 1 and the following equations, *e* indicates control error and is calculated as:

(1) e=SP−PV

The selected indices are calculated as follows (*N*—number of samples in a window):

- $\bar{\left|e\right|}$ Mean absolute control error
  (2) $$\bar{\left|e\right|}=\frac{1}{N}\Sigma_{i=1}^{N}\left|e\left(i\right)\right|$$
- $\bar{e^{2}}$ Mean squared control error
  (3) $$\bar{e^{2}}=\frac{1}{N}\Sigma_{i=1}^{N}e^{2}\left(i\right)$$
- $\sigma_{CV}^{2}$ Control signal variance
  (4) $$\sigma_{CV}^{2}=\frac{1}{N}\Sigma_{i=1}^{N}{\left(CV\left(i\right)-\bar{CV}\right)}^{2},$$
  where $\bar{CV}$ is the mean value of CV.
- $\bar{\left|\Delta CV\right|}$ Mean control signal difference between consecutive samples:
  (5) $$\bar{\left|\Delta CV\right|}=\frac{1}{N-1}\Sigma_{i=1}^{N-1}\left|CV\left(i+1\right)-CV\left(i\right)\right|$$
- $CV_{osc}$ Control signal oscillation (change in direction) count:
  (6) $$CV_{osc}=\frac{1}{N-1}\Sigma_{i=1}^{N-1}||\left\{i:\Delta CV\left(i\right)*\Delta CV\left(i+1\right)<0,i=1,\cdots ,N-1\right\}||,$$
  where ||x|| indicates the number of elements in *x*.
- Saturation index
  (7) $$sat=\frac{\Sigma_{i=1}^{N}t_{sat}}{N},\;t_{sat}=\left\{\begin{array}{cc} 0 & \mathrm{for}(CV\geq 10\%)\wedge (CV\leq 90\%)\\ 1 & \mathrm{for}(CV<10\%)\vee (CV>90\%) \end{array}\right.,$$

### 3.2. Controller Modelling

The indices described in this section are not control loop performance measures. However, they have been proposed to detect changes in the controller algorithm that may result from malicious actions. A more detailed description and modelling results for different types of controllers can be found in [15].

In order to determine the indices for each control loop, two models are trained, estimating the increment in the control signal ΔCV:

- The linear model is a linear regression model that takes the control error and lagged error values as inputs. The coefficients of the linear model allow the calculation of estimated PID controller settings. The model can only work for linear data—parts with regulator saturation are excluded from learning and prediction.
- The neural model is a non-linear model that takes control error and lagged error values and the control signal value as inputs. A model in the form of a multilayer perceptron was used. This model does not allow for controller settings estimation but can be used for controller types other than PID and data with controller saturation present.

Residuals of modelling errors are calculated as:

(8) $$r_{linear}=\Delta {\hat{CV}}_{linear}-\Delta CV,$$

where $\Delta {\hat{CV}}_{linear}$ is a linear model prediction and ΔCV is a real controller output.

(9) $$r_{nn}=\Delta {\hat{CV}}_{nn}-\Delta CV,$$

where $\Delta {\hat{CV}}_{nn}$ is a neural model prediction.

#### Controller Settings Estimation Form Linear Model

This section presents only equations for the PI controller used in the case study. Details for other controller types can be found in [15].

The following equations were based on a linear model of the ideal PI controller in incremental version:

(10) $$\Delta CV\left(k\right)=k_{p}\left(e\left(k\right)-e\left(k-1\right)+\frac{T_{s}}{T_{i}}e\left(k-1\right)\right),$$

where *k*—sample number, ΔCV(k)=CV(k)−CV(k−1), e(k)—control error, $k_{p}$—proportional gain, and $T_{i}$—integral time $T_{s}$—sampling time.

The inputs of the controller model are e(k) and e(k−1), and the model takes the form of:

(11) $$\Delta \hat{CV}\left(k\right)=a_{1}e\left(k\right)+a_{2}e\left(k-1\right)$$

where $\Delta \hat{CV}\left(k\right)$—model output, and $a_{1}$ and $a_{2}$—model coefficients. The coefficients are estimated using mean squared error minimisation.

The controller parameters can be calculated using the estimated linear model coefficients:

(12) $$k_{p}=a_{1},\;T_{i}=\frac{T_{s}}{\frac{a_{2}}{k_{p}}+1}.$$

### 3.3. Preprocessing

**Figure 3:**
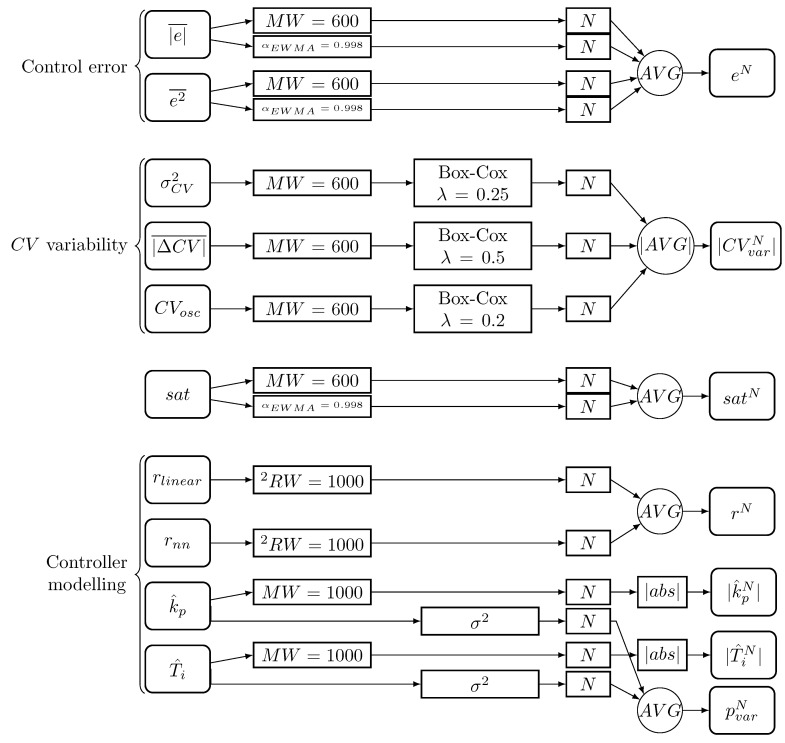
Preprocessing.

The signals considered are either a time series matched by sampling period to the controller processing period (e.g., $r_{linear}$) or statistics or estimates determined for a specific time window (e.g., $\bar{\left|e\right|}$ or ${\hat{T}}_{i}$). In addition, each indicator has a different range of values, making it challenging to combine them and select alarm limits. Therefore, the following preprocessing steps were used (Figure 3):

- Control loop performance indices are calculated in mowing windows (MW) and (if applicable) as exponentially weighted moving average (EWMA):
  - mowing windows are overlapping with an offset equal to half of the length of the moving window size
  - the exponentially weighted moving average of a time series {x(1),x(2),⋯,x(N)} is calculated as:
    (13) $$x_{EWMA}\left(k\right)=\alpha_{EWMA}x_{EWMA}\left(k-1\right)+\left(1-\alpha_{EWMA}\right)x\left(k\right),$$
    where $\alpha_{EWMA}$ is the smoothing factor.
- The controller modelling residuals $r_{linear}$ and $r_{nn}$ are squared (${}^{2}$) and averaged in moving window with an offset equal to 1 (RW).
- The time series related to controller variability ($\sigma_{CV}^{2}$, $\bar{\left|\Delta CV\right|}$, $CV_{osc}$) has non-normal character. They are transformed using Box-Cox transformation:
  (14) $$x_{Box-Cox}=\frac{x^{\lambda }-1}{\lambda },$$
  where λ is an exponent coefficient.
- All of the signals are normalised using a standard scaler (subtracting mean and dividing by standard deviation):
  (15) $$x^{N}=\frac{x-\bar{x}}{\sigma_{x}},$$
  where $\bar{x}$ and $\sigma_{x}$ denote the mean and standard deviation, respectively. Superscript ${}^{N}$ denotes the normalised value.
- To achieve higher robustness and interpretability, the signals are grouped into indices according to the process feature that they describe:
  - Control error increase $e^{N}=\mathrm{avg}\left({\bar{\left|e\right|}}_{MW}^{N},{\bar{\left|e\right|}}_{EWMA}^{N},{\bar{e^{2}}}_{MW}^{N},{\bar{e^{2}}}_{EWMA}^{N}\right)$
  - Change in CV variability $|CV_{var}{|}^{N}=\left|\mathrm{avg}\left({\left(\sigma_{CV}^{2}\right)}^{N},{\bar{\left|\Delta CV\right|}}^{N},CV_{osc}^{N}\right)\right|$
  - High CV saturation $sat^{N}=\mathrm{avg}\left(sat_{MW}^{N},sat_{EWMA}^{N}\right)$
  - High controller model prediction error $r^{N}=\mathrm{avg}\left(r_{liner}^{N},r_{nn}^{N}\right)$
  - Change in $k_{p}$ estimation $|{\hat{k}}_{p}^{N}|$
  - Change in $T_{i}$ estimation $|{\hat{T}}_{i}^{N}|$
  - High controller parameters estimation variability $p_{var}^{N}=\mathrm{avg}\left(\sigma_{kp}^{2},\sigma_{Ti}^{2}\right)$

After the preprocessing, we obtain the final set of signals:

(16) $$S=\{r^{N},|{\hat{k}}_{p}{|}^{N},|{\hat{T}}_{i}{|}^{N},|CV_{var}{|}^{N},sat^{N},e^{N},p_{var}^{N}\}$$

These signals are thresholded to obtain the set of binarised alarm signals (diagnostic signals):

(17) $$S=\{r^{A},|{\hat{k}}_{p}{|}^{A},|{\hat{T}}_{i}{|}^{A},|CV_{var}{|}^{A},sat^{A},e^{A},p_{var}^{A}\}$$

All of the preprocessing parameters (window sizes, smoothing factor $\alpha_{EWMA}$, exponent λ for Box-Cox transformation, and alarm thresholds) are tuned using only the data from regular system operation (without any faults or cyber-faults). Controller models (linear and neural) are also fitted to the data from the regular operation. The current values of controller parameters’ estimates ${\hat{k}}_{p}$ and ${\hat{T}}_{i}$ are calculated from a linear model estimated in a sliding window. However, the model used for calculating residual $r_{linear}$ is not updated.

### 3.4. Isolation Method

Our cyber-attack detection and isolation method is based on standard techniques used in the FDI community [4]. Fault detection and isolation are based on a set of diagnostic tests. Each *j*-th diagnostic test outputs a diagnostic signal $s_{j}$ indicating the result of the check. As a result of all the tests, we obtain the set of all diagnostic signals *S*:

(18) $$S=\{s_{j}:j=1,\cdots ,J\}.$$

To isolate the faults, it is necessary to know the relationship between the faults forming the set:

(19) $$F=\{f_{k}:k=1,\cdots ,K\}$$

and the values of the diagnostic signals. Expert knowledge about the fault-symptom relation can be described and archived in many different forms. A binary relation can be represented by: logic functions, diagnostic trees, a binary diagnostic matrix, or a set of rules [4]. In the case under consideration, these faults will be cyber-attack scenarios.

The most popular method of fault-symptom relation representation is a binary diagnostic matrix (Table 2). It is defined over the Cartesian product of *S* and *F*, so it specifies the relation:

(20) $$R_{FS}\subset F\times S.$$

The expression $<f_{k},s_{j}>\in R_{FS}$ means that diagnostic signal $s_{j}$ is sensitive to fault $f_{k}$. The occurrence of $f_{k}$ sets the value of $s_{j}$ to one, i.e., indicating a fault symptom. The matrix of this relation is called a binary diagnostic matrix (see the small example in Table 2). Each matrix entry is defined as follows:

(21) $$v_{jk}=\left\{\begin{array}{cc} 0 & \Leftrightarrow <f_{k},s_{j}>\notin R_{FS}\\ 1 & \Leftrightarrow <f_{k},s_{j}>\in R_{FS} \end{array}\right..$$

Matrix element $v_{jk}$ has a value of one if signal $s_{j}$ detects fault $f_{k}$, and zero otherwise. A fault signature is a column vector containing the values of the diagnostic signals for this fault:

(22) $$\left[\begin{array}{c} v_{1k}\\ v_{2k}\\ \cdots \\ v_{Jk} \end{array}\right],$$

where $v_{jk}\in \left\{0,1\right\},\forall j=1,\cdots ,J,k=1,\cdots ,K$.

Therefore, the columns of the binary diagnostic matrix (Table 2) correspond to fault signatures.

Given the binary diagnostic matrix and the actual values of diagnostic signals, we calculate the diagnosis by searching for the column of the binary diagnostic matrix with the maximal similarity to the values of diagnostic signals:

(23) $$f_{k}:k={\mathrm{argmax}}_{i}\Sigma_{j=0}^{J}\left(v_{ji}==s_{j}\right).$$

## 4. Case Study

**Figure 4:**
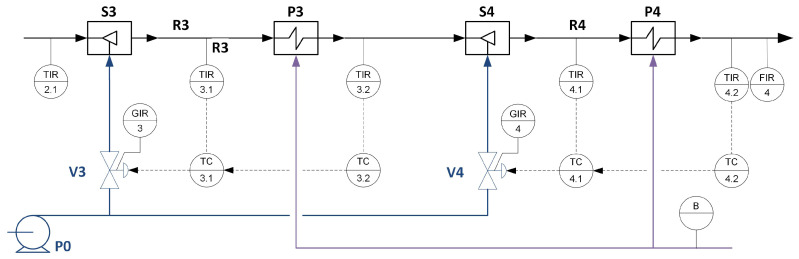
A schematic process diagram with designated control loops and process variables.

The developed modelling approach was tested for a superheater system under malicious interventions. The study used a model of superheaters of the third and fourth stages of the steam line in the boiler of the power unit. The process schematic diagram with available measured process variables is given in Figure 4.

The dynamic properties of the simulated process and its structure, including control loops and its parameters, were modelled based on the actual installation of a steam draught of the soda boiler in the paper company, which we collaborate with and which granted us access to process description, real data, and controller parameters, which were used during simulator elaboration. The simulator was implemented in the PExSim environment [54]. It is a computational and simulation environment developed at the Institute of Automatic Control and Robotics of the Warsaw University of Technology. It is visually similar to Simulink but is definitely simplified compared to it. It allows cyclic processing of signals according to the designed algorithm given in the form of a function block diagram. This is one of the possible simulation environments to be used. Choosing a specific simulation environment, i.e., its properties, do not have an impact on the conducted simulations and, consequently, on the results of the presented tests.

The modelled part of the process consists of the third and fourth steam line sections. In both, there are: attemperator, superheater, and cascade control systems—the main controller controls the temperature of the steam behind the superheater. The auxiliary controller controls the injection water valve and the temperature behind the cooler. The notation will be as follows: 3.1, 3.2—the auxiliary and main control loops of the third stage, respectively, 4.1, 4.2—the auxiliary and main control loops of the fourth stage, respectively. The available measurement variables directly related to the considered process with control loops, along with the determination of the ranges of variation and the operating points to which the model has been tuned based on data from the actual process, are characterised in Table 3.

The process was modelled in a simulation environment using a simplified linearised (at an operating point) model, reflecting the fundamental relationships between physical quantities. A specific variability of input quantities was assumed in the conducted experiments (temperature and steam flow at the inlet to the third stage and the contractual value of the fuel flow fed to the boiler) and cycles of the variability of setpoint (SP) values of the main controllers of the third and fourth stages: related changes in $SP_{3.2}$ and $SP_{4.2}$.

According to the place of introduction (Figure 2), cybernetic faults have been divided into the following types (marked with a superficial upper index):

- PV.P—PV modification at the process output (visible to the operator and controller),
- PV.C—PV modification at the controller input (visible only by the controller),
- SP.C—SP modification at the controller input (invisible to the operator),
- X.P—modification of the not-controlled process variable at the process output (visible to the operator and controller),
- CV.C—modification of CV at the controller output (visible to everyone),
- CV.P—modification of CV at the actuator input (visible only by the actuator),
- CV.UI—modification of CV at the monitoring system input (visible to the operator),
- C.x—modification (detailed described by x) of the controller’s operation,
- A.x—modification (detailed described by x) of the operation of the actuator.

**Figure 5:**
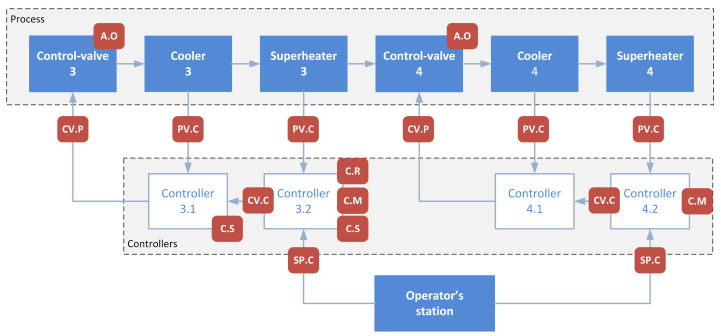
Places of influence on the process during the implementation of cyber-attacks (by the type of cybernetic fault).

The lower index indicates the control loop or specific component affected by the attack. In order to test different cyber-attack scenarios, the simulator includes the possibility of simulating cybernetic faults presented in Notations. The symbolic place of introducing the cybernetic faults performed during selected cyber-attacks is shown in Figure 5.

The number of possible attack scenarios is practically unlimited. The use of different scenarios was considered in terms of the conducted research. They were divided into groups depending on the attack component or group of signals. The particular groups can be characterised as follows:

1. attack on the controller (change in operating mode, change in parameters),
2. modification in set points,
3. modification in control variables,
4. modification in controlled variables,
5. attack on the actuator (blockage, modification in operation, changes in operating parameters).

Originally, we evaluated 27 cyber-attack scenarios of 12 types with different detailed parameters. However, we have decided to discuss, in this paper, only a subset of scenarios strictly related to controllers and most interesting to investigate. The particular cyber-attack scenarios selected for detailed research are presented in Table 4.

A specific variability of input signals, *B*, $F_{2}$, and $T_{2.1}$, symbolising disturbance, was assumed to generate learning and testing data. The value of each signal is the sum of four sinusoidal signals (with different parameters) and two random signals (with normal distribution but different gains), which are additionally processed by an inertial element to eliminate violent, physically unrealisable changes. The variability of individual signals is turned on or off at specified intervals every 6 h. Work scenarios for (a) constant SP values and (b) variable SP values according to a given scenario were also considered.

The dataset containing signals for all the considered scenarios and normal process operation is available at https://doi.org/10.5281/zenodo.7612269.

### Binary Diagnostic Matrix for Considered Scenarios

Based on the knowledge of the nature of the scenarios, an initial version of the binary diagnostic matrix presented in Table 5 was developed. It can be observed that some of the columns of the diagnostic matrix are the same, which means that the proposed diagnostic signals cannot isolate the given scenarios. Thus, to simplify, a reduced version of the binary diagnostic matrix was prepared, where the indistinguishable scenarios were combined. The reduced binary diagnostic matrix is presented in Table 6.

The linear model (and thus the controller parameter estimation) only works if the controller is not saturated, hence the blanks in the table for the CON-1 scenario.

The columns of the reduced binary diagnostic matrix (Table 6) can be interpreted as follows: cyber-attack CON-12 denotes an attack directed at the controller of a drastic nature (switching to manual mode, changing from normal to reverse). In this case, we observe a residuum of the controller model ($r^{A}$) as well as a deterioration in the quality of system operation (high control error).

The CON-3 scenario denotes changes in the controller (e.g., a change in the settings) but of a nature that does not entirely prevent the system’s operation. We observe an error in the controller model and changes in the settings estimation, but the control quality does not deteriorate drastically. In the case of a PID controller, more accurate information about changes can be obtained from the values of the estimated settings.

PVSP scenarios imply falsification of the values fed to the controller (PV or SP, respectively). In this case, we receive alarms about the regulator’s model error and the change in the settings estimate. However, in contrast to the CON-3 scenario, the settings estimates are inconsistent and have a high variance. The control error increases, but we do not observe a change in the character of the controller output (saturation or variance change) because the controller is operating correctly but on different values, than are recorded by the operator.

ACTCV scenarios represent attacks directed at the actuator or falsification of the CV value fed to the process, respectively. From the point of view of the controller models and control quality indicators, this is visible as a process change. The controller works correctly (the models show no deviation), but the regulation quality deteriorates (the control error increases), and the controller can enter a saturation zone. Note that changes in the process or actuator that the controller can compensate for will not be detectable, as will be demonstrated in the example scenario ${}_{A.1}^{ACT-2}$.

## 5. Results

This section will discuss the results obtained for the test scenarios.

Table 7 shows the results obtained without cyber-attacks. The attack starts in the middle of a given data file in each scenario. The data before the attacks were used to evaluate the performance in the normal state. Table 7 shows the results averaged over all scenarios. The columns show the subsequent control circuits. The rows show the averaged values of the alarm signals. The row %correct indicates the percentage of correct diagnoses (in this case, the correct diagnosis is always normal). The row %FPR presents the false positive alarm rate, which equals 1−%correct. We can see that the percentage of false alarms is low for individual diagnostic signals and resultant diagnoses, and the system works correctly in cases without cyber-attacks.

The actual ($k_{p}$ and $T_{i}$) and estimated (${\hat{k}}_{p}$ and ${\hat{T}}_{i}$) values of the controller settings are shown in Table 8. The columns show the subsequent scenarios. Note that some of the scenarios involve changing the settings. The estimates of the settings are close to the actual values and provide valuable diagnostic information in the case of attacks. The errors of the estimates are significant only for scenarios ${}_{A.1}^{SP-1}$ and ${}_{A.1}^{PV-1}$, where spurious signal values are fed into the controller. We can detect this situation based on the variance in the parameter estimate $p_{var}^{A}$.

The performance results during the attacks are presented by the control loop in Table 9, Table 10, Table 11 and Table 12. The columns show the subsequent scenarios. Note that each attack can affect from one to all control loops. The names of the scenarios in which a particular loop is affected have been bolded. When an attack does not affect a loop, the correct diagnosis is normal. The rows show the average values of the diagnostic signals during the attack. %correct denotes the percentage of correct diagnoses, detection denotes the percentage of attack detections (diagnoses other than normal), and diagnosis is the most frequent diagnosis. The tables are divided by a vertical line into a controller-directed attack part (left part) and a process-directed attack part (right part). The left-hand part demonstrates the proposed approach’s effectiveness in detecting and localising controller cyber-attacks. The right-hand section presents tests for process-directed attacks. The purpose of this part is to test the applicability and deficits of the proposed approach and to provide directions for further work.

For the scenarios considered, the fault isolation process involves two issues. One is to decide which control circuit is affected by the attack and what attack it is. It should be noted that an attack on one of the control circuits can change the operating conditions of the entire process, so it significantly increases the risk of false alarms in the other loops. However, it is most important to identify the loop that needs to be addressed first.

The results of the cyber-attack isolation are shown in Table 13 and Table 14. Table 15 indicates which attacks affect which loop. This provides a template for the correct locations regarding control loop selection. Table 13 shows the results obtained regarding control loop isolation. A value of 1 indicates that the diagnosis differed from the normal state in the respective loop over 50% of the time. Incorrect values are marked in red. We can observe that scenario ${}_{A.1}^{ACT-2}$ was not detected in any control loop—the case of this scenario will be analysed in detail in the plots. All other scenarios were detected. For the controller scenarios, the isolation in terms of the control circuit is precise (one false alarm for loop 4.1). False alarms for the scenarios ${}_{A.1}^{CV-1}$ and ${}_{A.1}^{ACT-1}$ appear on the left-hand side of the table. These are due to the significant impact of the deterioration in the main circuit on the auxiliary circuit (${}_{A.1}^{CV-1}$) and the impact of the deterioration in stage 3 on the operation of stage 4 (${}_{A.1}^{ACT-1}$).

**Figure 6:**
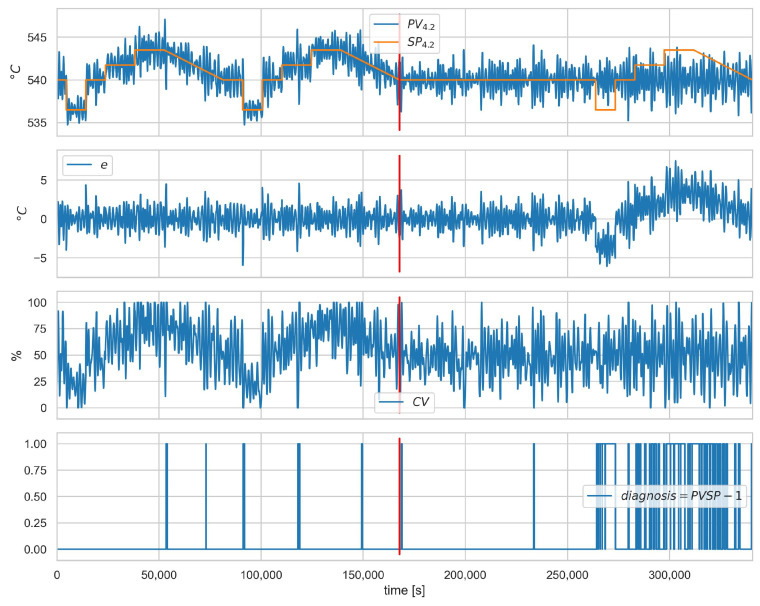
Scenario ${}_{A.1}^{SP-1}$, loop 4.2, values of PV, SP, *e*, and CV. The lowest plot shows the presence of the correct diagnosis SPPV. Red vertical line indicates the beginning of cyber-attack.

**Figure 7:**
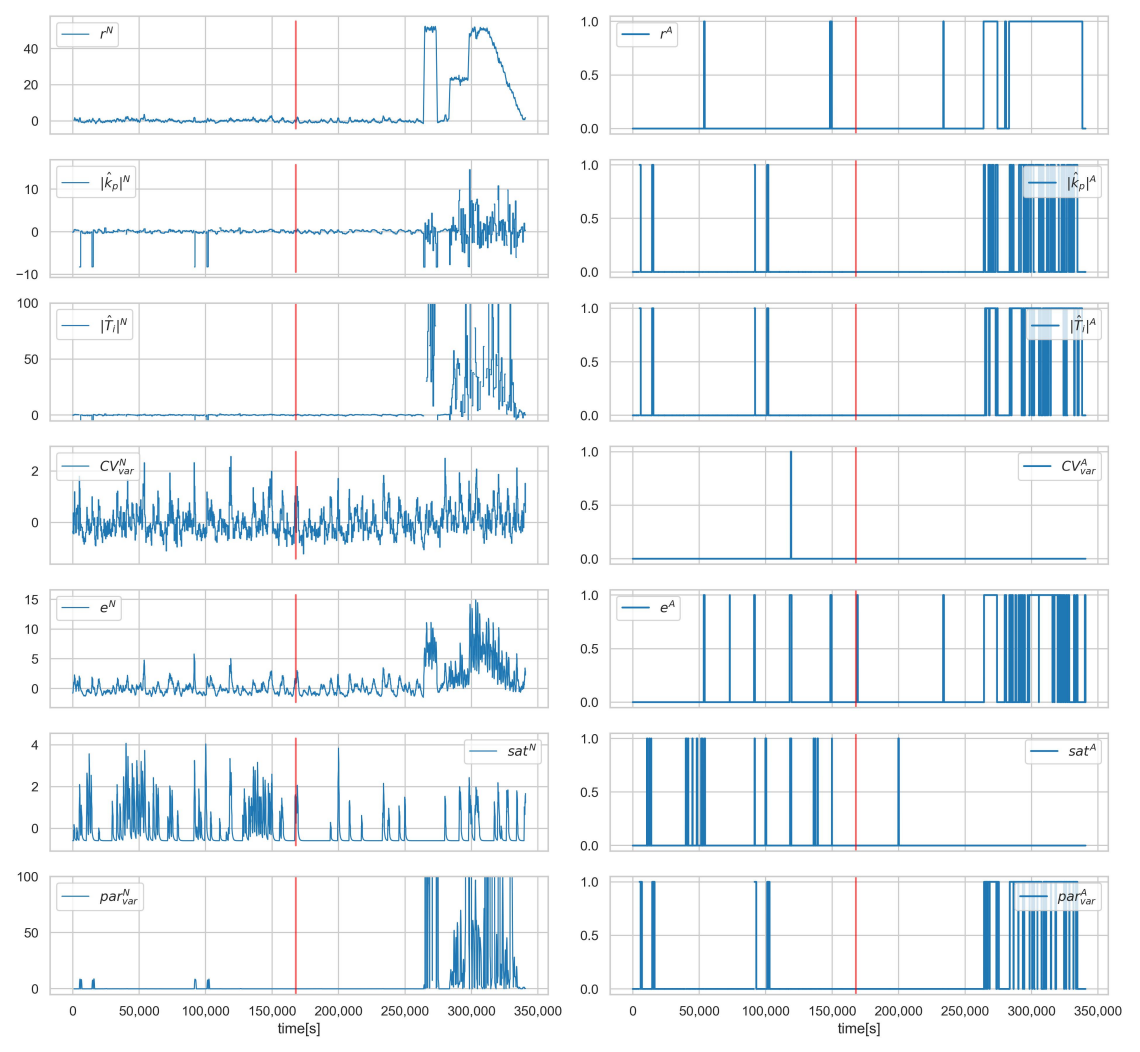
Scenario ${}_{A.1}^{SP-1}$, loop 4.2, values of normalised signals (**left**), and binarised diagnostic signals (**right**). Red vertical line indicates the beginning of cyber-attack.

The results of the isolation in terms of the scenario are shown in Table 14. Again, attacks targeting the controller operation were correctly identified (one false alarm for loop 4.1). Scenario ${}_{A.1}^{SP-1}$ involves substituting the value of SP for a fixed value and can only be correctly detected and recognised when the actual value of SP differs from the provided fixed value. This is explained in more detail in the graphs in (Figure 6 and Figure 7). The two attacked loops indicate the correct diagnosis for scenario ${}_{A.1}^{ACT-1}$. Scenario ${}_{A.1}^{CV-1}$ was correctly detected but is mistaken for a CON-12 diagnosis.

**Figure 8:**
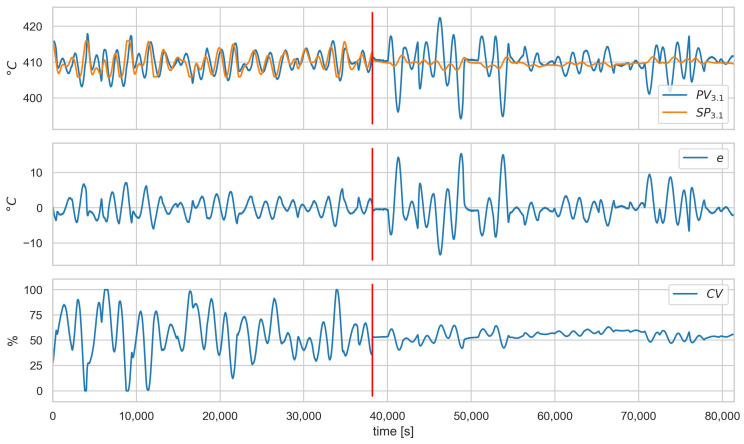
Scenario ${}_{B.1}^{CON-3}$, loop 3.1, values of PV, SP, *e*, and CV. Red vertical line indicates the beginning of cyber-attack.

**Figure 9:**
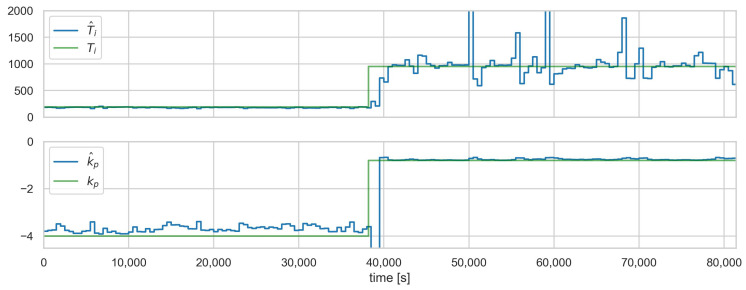
Scenario ${}_{B.1}^{CON-3}$, loop 3.1, and controller settings estimates (blue—estimates, green—actual values).

**Figure 10:**
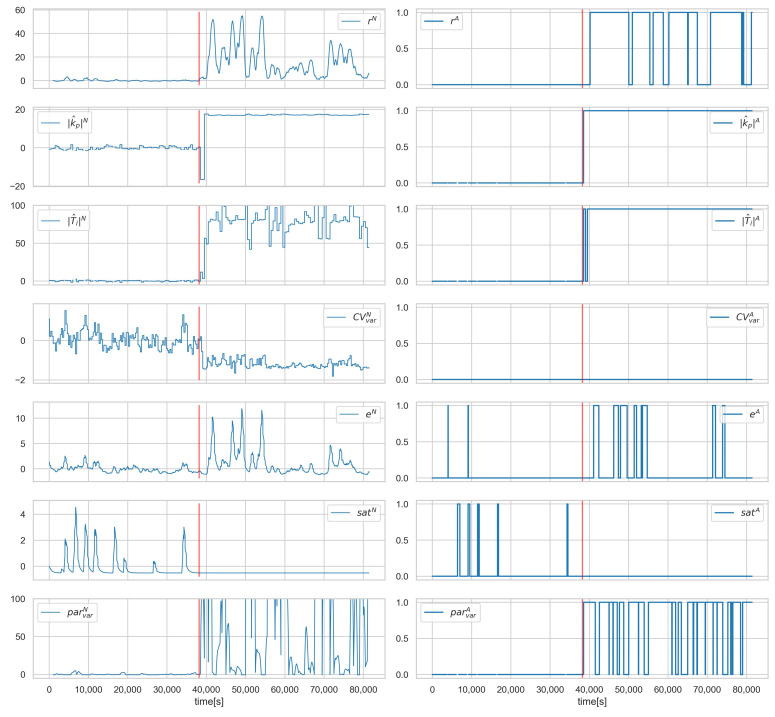
Scenario ${}_{B.1}^{CON-3}$, loop 3.1, values of normalised signals (**left**), and binarised diagnostic signals (**right**). Red vertical line indicates the beginning of cyber-attack.

Figure 8, Figure 9 and Figure 10 show the runs for scenario ${}_{B.1}^{CON-3}$ and control loop 3.1. This scenario involves changing the controller settings to less aggressive. Figure 8 shows PV, SP, *e*, and CV, respectively. The red vertical line indicates the moment of attack. Figure 9 shows the estimated and actual parameter values. We can see that the estimates are close to the actual values. The values of the diagnostic signals are shown in Figure 10. These signals behave as predicted—we observe alarms for the model error and the controller parameter estimates. Intermittent alarms indicate an increase in the control deviation, which is consistent with the actual state.

Figure 6 and Figure 7 show the runs for scenario ${}_{A.1}^{SP-1}$ and control loop 4.2. Figure 6 shows the values of the variables and, in the lower plot, the presence of the correct diagnosis SPPV. This scenario consists of substituting the SP value fed to the controller. Symptoms of this attack can only be observed when the actual SP value deviates from the falsified one, which is observed from about 260,000 s. The same dependence can be observed for the diagnostic signals (Figure 7).

**Figure 11:**
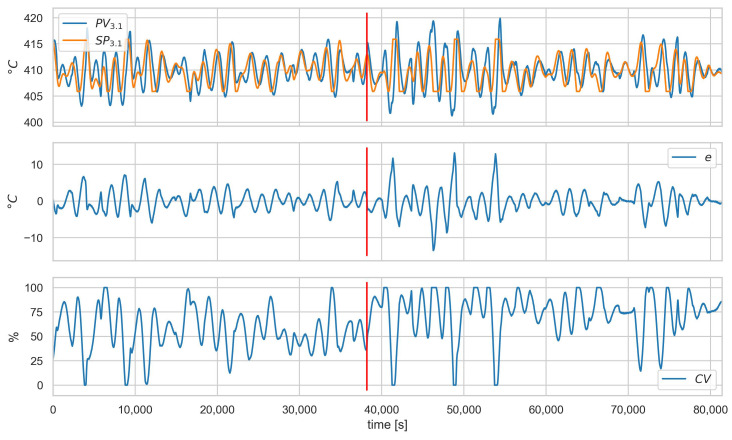
Scenario ${}_{A.1}^{ACT-2}$, loop 3.1, values of PV, SP, *e*, and CV. Red vertical line indicates the beginning of cyber-attack.

**Figure 12:**
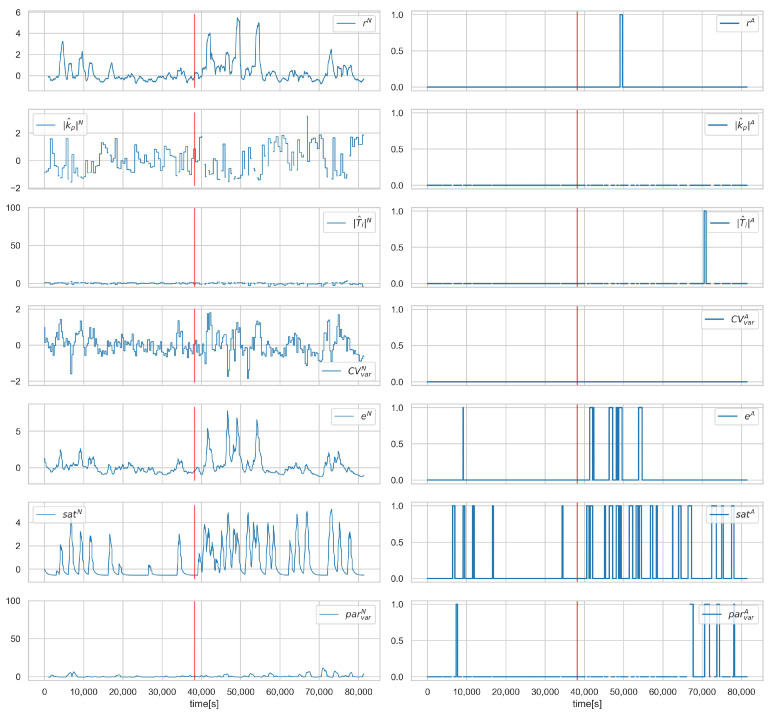
Scenario ${}_{A.1}^{ACT-2}$, loop 3.1, values of normalised signals (**left**), and binarised diagnostic signals (**right**). Red vertical line indicates the beginning of cyber-attack.

Figure 11 and Figure 12 show the values of the process and alarm signals, respectively, for scenario ${}_{A.1}^{ACT-2}$ and loop 3.1. In this scenario, the control valve is attacked (its closure ratio is changed by 20 %). We can observe, in Figure 11, that this causes a change in the average value of CV. However, the control system can compensate for the attack, and we do not observe a deterioration in the quality of the control. This can also be seen in the diagnostic signals (Figure 12)—only a slight increase in the saturation index $sat^{A}$ is visible. Since the controller is working correctly and there is no evident deterioration of the control quality, this scenario is impossible to detect in the proposed solution. In the authors’ opinion, this problem should be solved by introducing process models into the system in further development.

### Sensitivity Analysis

As part of the sensitivity analysis, the robustness of the proposed method to process changes and changes in the nature of cyber-faults was tested. For this purpose, a baseline scenario ${}_{A.1}^{CON-3}$ containing two cyber-faults, $cf_{3.1}^{C.S}$ and $cf_{3.2}^{C.S}$, involving a change in controller settings to a more aggressive one was modified. Modifications of 25%, 50%, and 150% of the baseline change were applied. The specific values of the controller settings are given in Table 16. The effect of varying the amplitude of the disturbance was also tested. The disturbance was taking 50% and 150% of the baseline value (50%dist and 150%dist scenarios, respectively).

No modifications were made to the method or parameters. The same neural networks and linear models were used in the tests as in the earlier experiments. The models were trained with a baseline level of noise. The data normalisation factors and alarm thresholds were not changed.

The results are presented in Table 17. For each scenario, the percentage of detection, %detection, and percentage of correct diagnoses, %correct, are shown. The results are shown for each loop. In this scenario, loops 3.1 and 3.2 are attacked, and these column names have been bolded. We can see that the isolation within the control loop continues to be very precise. Missed detections can be observed for small settings changes (25%). For significant disturbances, few false alarms appear for the 4.1 and 4.2 loops. For substantial settings changes (150%), the cyber-attack type isolation starts to indicate the CON−12 scenario. This scenario means drastic changes in the controller preventing effective regulation, and this classification for significant settings changes can be considered correct.

The results of estimating the controller settings under different conditions are shown in Table 16. The settings are estimated correctly with an accuracy close to the baseline scenarios.

From the tests, it can be concluded that the proposed method has some robustness. Of course, detecting small changes in the presence of significant disturbances will be difficult. Further, a change in the system’s operating conditions (change in the nature of the inputs, severity of the disturbances) may lead to the need for retraining the models and re-tuning the normalisation factors and alarm limits. Both of these processes can be carried out automatically once new, representative data have been acquired.

## 6. Discussion

This work presents controller modelling and control loop performance indicators as tools for detecting and isolating cyber-attacks. Controller models allow the detection of changes in controller performance and settings (in the case of PID controllers). Control quality indicators allow an overall assessment of the performance of control circuits and the detection of deterioration in control quality.

The selection of suitable control quality indices and the preprocessing and combining of signals to obtain a set of interpretable indices are presented.

The operation of the concept was tested on a superheater system. The system’s overall performance should be considered a valuable indication for the process operator. In regular operation, the percentage of false alarms is low at 0.48%. All attacks except ${}_{A.1}^{ACT-2}$ were detected (the issue of not being able to detect attack ${}_{A.1}^{ACT-2}$ is further detailed in Figure 11 and Figure 12).

The system performs very well in attacks directly targeting the controller’s operation. All attacks were correctly detected. The detection percentage in the attacked loops is 99.09% (attacks relating to a given loop are indicated in bold in Table 9, Table 10, Table 11 and Table 12). The isolation of the control loop is accurate (one false alarm for loop 4.1 in scenario ${}_{B.1}^{CON-1}$). The isolation in terms of scenarios is also accurate (the same false alarm for loop 4.1 in scenario ${}_{B.1}^{CON-1}$), and interpretable diagnostic signals allow the nature of the attack (such as the nature of the setting change) to be identified more accurately. The system’s overall accuracy (percentage of correct diagnoses) is 85.05%.

In terms of other attacks, the system provides valuable indications of system changes—all attacks except ${}_{A.1}^{ACT-2}$ have been detected. The attacked circuits are mainly indicated as the source of the problem (84.99% percentage of detection for an attack except for ${}_{A.1}^{ACT-2}$). However, the change in operating conditions also increases the occurrence of alarms in the remaining control loops, even if they are not directly affected by the attack. Scenario ${}_{A.1}^{ACT-2}$ shows that process models are needed to detect attacks that the controller action can mask.

It should be noted that indicators calculated in a sliding window (such as PID controller parameter estimates) inevitably introduce fault detection and isolation delays. Using process models is a way to obtain indicators that react faster to anomalies.

## 7. Conclusions

In advanced diagnostics of industrial control systems (ICS) performed automatically, faults and cyber-attacks should be detected and isolated. Compared to the classic FDI approach, the area of diagnostic activities is, therefore, extended and should include not only process apparatus, measurements, and actuators but also units implementing control algorithms. Controller models and control loop performance indicators can be used effectively to detect cyber-attacks and faults that manifest in changes to control systems’ operation. The isolation of controller faults and cyber-attacks can be performed using inference methods based on the binary diagnostic matrix. The case study showed that correct identification of malfunctioning control loops and introduced cyber-attack scenarios could be achieved. Experimental verification should be carried out on a process simulator allowing the introduction of both faults and cyber-attacks.

It is planned to test additional scenarios in this way. The conduct of industrial tests is much more problematic due to limitations on the possibility of introducing faults and cyber-attacks. In addition, consent for such experiments will not be given by company management due to the risks of such research. In this situation, industrial verification can take place after thorough simulation verification during a pilot implementation on a real installation.

The direction of further work is to develop an integrated approach for fault and cyber-attack detection and isolation in ICS, which should additionally include detection based on process and actuator models and the isolation of cyber-attacks and faults based not only on binary residual evaluation but also on trivalent evaluation.

## Figures and Tables

**Table 1:** Considered loop performance indicators (${}^{*}$ points to the indicators selected for use in the final solution).

Category	Label	Index Description
Basic statistics	$\bar{e}$	Mean control error
	$\bar{\left|e\right|}$	Mean absolute control error ${}^{*}$
	$\bar{e^{2}}$	Mean squared control error ${}^{*}$
	$\sigma_{CV}^{2}$	Control signal variance ${}^{*}$
	$\bar{\left|\Delta CV\right|}$	Mean control signal difference ${}^{*}$
	$CV_{osc}$	Control signal oscillation count ${}^{*}$
Controller benchmarking	$\eta_{MV}$	Harris index [11]
Response to a step change in SP	$T_{set}$	Settling time
	α	Overshoot
	$T_{set}^{*}$	Normalised settling time [51]
	$IAE_{d}$	Normalised integral absolute error [51]
Oscillation detection	$n_{I}$	Regularity of large values of IAE [52]
	*r*	Regularity of sign changes of autocorrelation function [53]
Nonlinearity detection	sat	Saturation index ${}^{*}$
Controller modelling	$r_{linear}$	Linear model residual ${}^{*}$
	${\hat{k}}_{p}$	Proportional gain estimation ${}^{*}$
	${\hat{T}}_{i}$	Integral time estimation ${}^{*}$
	$T_{d}$	Derivative time estimation
	$r_{nn}$	Neural model residual ${}^{*}$

**Table 2:** Binary matrix of $R_{XS}$ relation.

S\F	$f_{1}$	$f_{2}$	$f_{3}$	$f_{4}$
$s_{1}$	1	0	1	0
$s_{2}$	0	0	1	1
$s_{3}$	0	1	0	1

**Table 3:** Available process variables.

Section/Control Loop	Variable	Description	Units	Range	Working Point
-	*B*	Fuel inflow to the boiler (contractual variable)	%	0–100	85
-	$F_{2}$	Steam inflow to Section 3	kg/s	30–140	117.4
4	$F_{4}$	Steam flow in superheater 4 (at boiler outlet)	kg/s	30–140	120
3	$G_{3}$	Position of the injection valve of attemperator 3	%	0–100	55.4
3	$T_{2.1}$	Steam temperature at the inlet to the attemperator 3	C	395–445	420
3/TC3.1	$PV_{3.1}$	Steam temperature at the outlet from the attemperator 3	C	385–435	410
3/TC3.1	$SP_{3.1}$	Set point of the steam temperature at the outlet from the attemperator 3	C	-	410
3/TC3.1	$CV_{3.1}$	Control signal of temperature controller at the outlet of the attemperator 3	%	0–100	55.4
3/TC3.2	$PV_{3.2}$	Steam temperature at the outlet of superheater 3	C	465–515	492
3/TC3.2	$SP_{3.2}$	Set point of the steam temperature at the outlet of superheater 3	C	-	492
3/TC3.2	$CV_{3.2}$	Control signal of temperature controller at the outlet of the superheater 3	C	0–100	41
4	$G_{4}$	Position of the injection valve of attemperator 4	%	0–100	60.1
4/TC3.1	$PV_{4.1}$	Steam temperature at the outlet from the attemperator 4	C	455–505	479
4/TC3.1	$SP_{4.1}$	Set point of the steam temperature at the outlet from the attemperator 4	C	-	479
4/TC3.1	$CV_{4.1}$	Control signal of temperature controller at the outlet of the attemperator 4	%	0–100	60.1
4/TC3.2	$PV_{4.2}$	Steam temperature at the outlet of superheater 4	C	515–565	540
4/TC3.2	$SP_{4.2}$	Set point of the steam temperature at the outlet of superheater 4	C	-	540
4/TC3.2	$CV_{4.2}$	Control signal of temperature controller at the outlet of the superheater 4	C	0–100	52.69

**Table 4:** The set of cyber-attack scenarios (CAS). Each attack starts at $t_{0}^{CA}=43,200$ (s).

Type	Loop	CAS	Description	Used Cybernetic Faults
1	4.2	${}_{A.1}^{CON-1}$	Change in the operation mode (to “manual”) of the main controller 4.2 and setting a constant value of CV ($CV_{4.2}=100\%$)	$cf_{4.2}^{C.M}$, $cf_{4.2}^{CV.C}$
1	3.2, 4.2	${}_{B.1}^{CON-1}$	Change in the operation mode (to “manual”) of the main controllers 3.2 and 4.2 to “manual” and setting a constant values of CVs ($CV_{3.2}=100\%$, $CV_{4.2}=0\%$)	$cf_{3.2}^{C.M}$, $cf_{3.2}^{CV.C}$, $cf_{4.2}^{C.M}$, $cf_{4.2}^{CV.C}$
1	3.2	${}_{A.1}^{CON-2}$	Change in the operation mode of the main controller 3.2 to “reverse”—positive feedback	$cf_{3.2}^{C.R}$
1	3.1, 3.2	${}_{A.1}^{CON-3}$	Change in the settings of the main 3.2 ($k_{p}^{3.2}=5k_{p}^{3.2}$, $T_{i}^{3.2}=0.2T_{i}^{3.2}$) and auxiliary 3.1 ($k_{p}^{3.1}=5k_{p}^{3.1}$, $T_{i}^{3.1}=0.2T_{i}^{3.1}$) controllers to more aggressive	$cf_{3.1}^{C.S}$, $cf_{3.2}^{C.S}$
1	3.1, 3.2	${}_{B.1}^{CON-3}$	Change in the settings ($k_{p}$, $T_{i}$) of the main 3.2 ($k_{p}^{3.2}=0.2k_{p}^{3.2}$, $T_{i}^{3.2}=5T_{i}^{3.2}$) and auxiliary 3.1 ($k_{p}^{3.1}=0.2k_{p}^{3.1}$, $T_{i}^{3.1}=5T_{i}^{3.1}$) controllers to more passive	$cf_{3.1}^{C.S}$, $cf_{3.2}^{C.S}$
2	3.2, 4.2	${}_{A.1}^{SP-1}$	Setting a constant value of setpoint for main controllers 3.2 ($SP_{3.2}\left(k\right)=SP_{3.2}\left(t_{0}^{CA}\right)$) and 4.2 ($SP_{4.2}\left(k\right)=SP_{4.2}\left(t_{0}^{CA}\right)$)	$cf_{3.2}^{SP.C}$, $cf_{4.2}^{SP.C}$
3	3.1, 4.1	${}_{A.1}^{CV-1}$	Providing the value of the control signal from auxiliary controllers 3.1 and 4.1 to the process from the looped history (length of the history window: 6 (h))	$cf_{3.1}^{CV.P}$, $cf_{4.1}^{CV.P}$
4	3.1, 3.2, 4.1, 4.2	${}_{A.1}^{PV-1}$	Modification of the controlled process values PV for main and auxiliary controllers of stages 3 ($PV_{3.1}=PV_{3.1}-5$(C), $PV_{3.2}=PV_{3.2}-5$(C)) and 4 ($PV_{4.1}=PV_{4.1}-10$(C), $PV_{4.2}=PV_{4.2}-10$(C))	$cf_{3.1}^{PV.C}$, $cf_{3.2}^{PV.C}$, $cf_{4.1}^{PV.C}$, $cf_{4.2}^{PV.C}$
5	3.1	${}_{A.1}^{ACT-1}$	Taking control of the operation of the stage 3 actuator—lock in fixed position ($G_{3}=10\left[\%\right]$)	$cf_{V_{3}}^{A.O}$
5	3.1, 4.1	${}_{A.1}^{ACT-2}$	Reducing the degree of opening of the actuators of stages 3 ($G_{3}=G_{3}-20\left[\%\right]$) and 4 ($G_{3}=G_{3}+20\left[\%\right]$)	$cf_{V_{3}}^{A.O}$, $cf_{V_{4}}^{A.O}$

**Table 5:** Initial binary diagnostic matrix.

	CON-1	CON-2	CON-3	*SP*	*CV*	ACT	*PV*	Normal
$r^{A}$	1	1	1	1	0	0	1	0
$|{\hat{k}}_{p}{|}^{A}$		1	1	1	0	0	1	0
$|{\hat{T}}_{i}{|}^{A}$		0	1	1	0	0	1	0
$|CV_{var}{|}^{A}$	1	1	0	0	1	1	0	0
$e^{A}$	1	1	0	1	1	1	1	0
$sat^{A}$	1	1	0	0	1	1	0	0
$par_{var}^{A}$		0	0	1	0	0	1	0

**Table 6:** Reduced binary diagnostic matrix.

	CON-12	CON-3	PVSP	ACTCV	Normal
$r^{A}$	1	1	1	0	0
$|{\hat{k}}_{p}{|}^{A}$	1	1	1	0	0
$|{\hat{T}}_{i}{|}^{A}$	0	1	1	0	0
$|CV_{var}{|}^{A}$	1	0	0	1	0
$e^{A}$	1	0	1	1	0
$sat^{A}$	1	0	0	1	0
$par_{var}^{A}$	0	0	1	0	0

**Table 7:** The values of diagnostic signals during normal operation (each column shows one control loop).

	3.1	3.2	4.1	4.2
$r^{A}$	0.02	0.00	0.01	0.00
$|k_{p}{|}^{A}$	0.00	0.02	0.00	0.01
$|T_{i}{|}^{A}$	0.01	0.02	0.00	0.03
$|CV_{var}{|}^{A}$	0.00	0.00	0.00	0.00
$e^{A}$	0.00	0.00	0.00	0.00
$sat^{A}$	0.04	0.00	0.00	0.00
$p_{var}^{A}$	0.02	0.01	0.00	0.03
%correct	99.58	99.32	99.90	99.29
%FPR	0.42	0.68	0.10	0.71

**Table 8:** Settings estimation.

		${}_{A.1}^{\mathrm{CON}-1}$	${}_{B.1}^{\mathrm{CON}-1}$	${}_{A.1}^{\mathrm{CON}-2}$	${}_{A.1}^{\mathrm{CON}-3}$	${}_{B.1}^{\mathrm{CON}-3}$	${}_{A.1}^{\mathrm{SP}-1}$	${}_{A.1}^{\mathrm{CV}-1}$	${}_{A.1}^{\mathrm{ACT}-1}$	${}_{A.1}^{\mathrm{ACT}-2}$	${}_{A.1}^{\mathrm{PV}-1}$	Normal
3.1	$k_{p}$	−4	−4	−4	−20	−1	−4	−4	−4	−4	−4	−4
	${\hat{k}}_{p}$	−3.68	−3.71	−3.65	−13.02	−0.91	−3.64	−3.76	−3.98	−3.65	−4.51	−3.68
	$T_{i}$	190	190	190	190	190	38	190	190	190	190	190
	${\hat{T}}_{i}$	177.31	178.28	173.30	39.34	993.61	175.06	176.51	192.97	172.98	312.39	177.01
3.2	$k_{p}$	6	−	−6	30	1.2	6	6	6	6	6	6
	${\hat{k}}_{p}$	5.89	5.52	−5.56	32.01	1.36	6.53	5.81	5.86	5.87	7.91	5.87
	$T_{i}$	190	−	190	38	950	190	190	190	190	190	190
	${\hat{T}}_{i}$	188.13	180.56	184.30	50.41	718.89	225.20	180.90	183.99	186.93	6581.89	187.20
4.1	$k_{p}$	−5	−5	−5	−5	−5	−5	−5	−5	−5	−5	−5
	${\hat{k}}_{p}$	−4.35	−5.02	−4.32	−4.51	−4.45	−4.41	−4.82	−4.58	−4.49	−5.28	−4.41
	$T_{i}$	170	170	170	170	170	170	170	170	170	170	170
	${\hat{T}}_{i}$	148.97	181.00	148.67	160.45	152.14	151.58	163.05	150.76	154.25	−42996	151.83
4.2	$k_{p}$	5	5	5	5	5	5	5	5	5	5	5
	${\hat{k}}_{p}$	4.74	4.65	4.82	4.76	4.89	4.89	4.83	4.93	4.89	5.37	4.81
	$T_{i}$	90	90	90	90	90	90	90	90	90	90	90
	${\hat{T}}_{i}$	88.42	79.29	87.15	86.80	88.61	99.92	86.07	88.89	88.82	8995.09	87.25

**Table 9:** The values of diagnostic signals in loop 3.1 during attacks. Bolded column names indicate scenarios affecting this loop.

	${}_{A.1}^{CON-1}$	${}_{B.1}^{CON-1}$	${}_{A.1}^{CON-2}$	${}_{A.1}^{\mathrm{CON}-3}$	${}_{B.1}^{\mathrm{CON}-3}$	${}_{A.1}^{SP-1}$	${}_{A.1}^{\mathrm{CV}-1}$	${}_{A.1}^{\mathrm{ACT}-1}$	${}_{A.1}^{\mathrm{ACT}-2}$	${}_{A.1}^{\mathrm{PV}-1}$
$r^{A}$	0.08	0.15	0.06	1.00	0.88	0.03	0.54	0.06	0.07	1.00
$|k_{p}{|}^{A}$	0.00	0.00	0.04	0.99	0.99	0.02	0.00	0.00	0.00	0.65
$|T_{i}{|}^{A}$	0.04	0.10	0.13	0.99	0.99	0.04	0.14	0.00	0.10	0.74
$|CV_{var}{|}^{A}$	0.00	0.00	0.00	0.65	0.00	0.00	0.17	0.74	0.00	0.00
$e^{A}$	0.08	0.00	0.04	0.01	0.18	0.03	0.61	0.99	0.09	0.46
$sat^{A}$	0.07	0.17	0.11	0.19	0.00	0.04	0.78	0.93	0.23	0.26
$p_{var}^{A}$	0.03	0.18	0.17	0.54	0.83	0.04	0.20	0.00	0.18	0.97
%correct	92.49	88.66	93.84	94.69	88.16	97.79	19.25	90.43	2.78	42.84
%detection	7.51	11.34	6.16	99.34	99.31	2.21	71.40	96.01	8.13	93.89
diagnosis	normal	normal	normal	CON-3	CON-3	normal	CON-12	ACTCV	normal	CON-3

**Table 10:** The values of diagnostic signals in loop 3.2 during attacks. Bolded column names indicate scenarios affecting this loop.

	${}_{A.1}^{CON-1}$	${}_{B.1}^{\mathrm{CON}-1}$	${}_{A.1}^{\mathrm{CON}-2}$	${}_{A.1}^{\mathrm{CON}-3}$	${}_{B.1}^{\mathrm{CON}-3}$	${}_{A.1}^{\mathrm{SP}-1}$	${}_{A.1}^{CV-1}$	${}_{A.1}^{\mathrm{ACT}-1}$	${}_{A.1}^{\mathrm{ACT}-2}$	${}_{A.1}^{\mathrm{PV}-1}$
$r^{A}$	0.08	0.99	0.99	0.99	0.70	0.62	0.31	0.25	0.10	1.00
$|k_{p}{|}^{A}$	0.00	NaN	0.85	1.00	0.99	0.20	0.12	0.00	0.03	0.80
$|T_{i}{|}^{A}$	0.01	NaN	0.28	1.00	0.98	0.52	0.16	0.19	0.03	0.75
$|CV_{var}{|}^{A}$	0.00	0.98	0.81	0.56	0.00	0.00	0.12	0.78	0.00	0.00
$e^{A}$	0.09	0.95	0.65	0.00	0.21	0.28	0.39	0.98	0.12	0.59
$sat^{A}$	0.02	0.99	0.92	0.66	0.00	0.00	0.42	0.88	0.02	0.01
$params_{var}^{A}$	0.00	NaN	0.48	0.25	0.73	0.46	0.23	0.22	0.02	0.99
%correct	91.52	97.92	85.88	68.97	85.23	23.03	56.10	71.43	1.54	58.53
%detection	8.48	98.95	99.31	99.03	98.15	57.89	43.90	96.76	11.11	99.25
diagnosis	normal	CON-12	CON-12	CON-3	CON-3	normal	normal	ACTCV	normal	PVSP

**Table 11:** The values of diagnostic signals in loop 4.1 during attacks. Bolded column names indicate scenarios affecting this loop.

	${}_{A.1}^{CON-1}$	${}_{B.1}^{CON-1}$	${}_{A.1}^{CON-2}$	${}_{A.1}^{CON-3}$	${}_{B.1}^{CON-3}$	${}_{A.1}^{SP-1}$	${}_{A.1}^{\mathrm{CV}-1}$	${}_{A.1}^{ACT-1}$	${}_{A.1}^{\mathrm{ACT}-2}$	${}_{A.1}^{\mathrm{PV}-1}$
$r^{A}$	0.00	1.00	0.11	0.00	0.19	0.02	0.92	0.89	0.12	1.00
$|k_{p}{|}^{A}$	0.02	0.15	0.01	0.00	0.01	0.00	0.67	0.12	0.00	0.70
$|T_{i}{|}^{A}$	0.02	0.15	0.01	0.00	0.02	0.01	0.23	0.12	0.00	0.72
$|CV_{var}{|}^{A}$	0.00	0.85	0.09	0.00	0.00	0.00	0.33	0.72	0.00	0.01
$e^{A}$	0.00	0.93	0.17	0.00	0.19	0.03	0.97	0.98	0.16	0.99
$sat^{A}$	0.02	0.98	0.20	0.00	0.15	0.01	0.92	0.91	0.04	0.29
$params_{var}^{A}$	0.06	0.00	0.02	0.00	0.02	0.00	0.15	0.31	0.01	1.00
%correct	97.69	0.69	82.94	100.00	83.50	98.33	7.17	3.06	1.12	77.14
%detection	2.31	99.31	17.06	0.00	16.50	1.67	98.60	96.94	7.58	99.69
diagnosis	normal	CON-12	normal	normal	normal	normal	CON-12	CON-12	normal	PVSP

**Table 12:** The values of diagnostic signals in loop 4.2 during attacks. Bolded column names indicate scenarios affecting this loop.

	${}_{A.1}^{\mathrm{CON}-1}$	${}_{B.1}^{\mathrm{CON}-1}$	${}_{A.1}^{CON-2}$	${}_{A.1}^{CON-3}$	${}_{B.1}^{CON-3}$	${}_{A.1}^{\mathrm{SP}-1}$	${}_{A.1}^{CV-1}$	${}_{A.1}^{ACT-1}$	${}_{A.1}^{\mathrm{ACT}-2}$	${}_{A.1}^{\mathrm{PV}-1}$
$r^{A}$	1.00	0.74	0.06	0.00	0.11	0.39	0.68	0.19	0.08	1.00
$|k_{p}{|}^{A}$	0.00	0.00	0.00	0.00	0.00	0.16	0.03	0.04	0.00	0.89
$|T_{i}{|}^{A}$	0.00	0.00	0.02	0.00	0.00	0.28	0.12	0.09	0.00	0.67
$|CV_{var}{|}^{A}$	0.98	0.98	0.03	0.00	0.01	0.00	0.27	0.55	0.00	0.00
$e^{A}$	0.99	0.31	0.08	0.00	0.12	0.26	0.82	0.88	0.10	0.99
$sat^{A}$	0.99	0.99	0.16	0.00	0.16	0.00	0.88	0.93	0.12	0.19
$params_{var}^{A}$	0.00	0.00	0.03	0.03	0.03	0.32	0.26	0.14	0.04	1.00
%correct	98.61	73.00	89.32	100.00	87.92	22.38	13.96	9.67	1.59	87.29
%detection	99.52	99.09	10.68	0.00	12.08	36.53	86.04	90.33	7.55	99.88
diagnosis	CON-12	CON-12	normal	normal	normal	normal	CON-12	ACTCV	normal	PVSP

**Table 13:** Attack detection results for each loop. Red color indicates incorrect values.

	${}_{A.1}^{\mathrm{CON}-1}$	${}_{B.1}^{\mathrm{CON}-1}$	${}_{A.1}^{\mathrm{CON}-2}$	${}_{A.1}^{\mathrm{CON}-3}$	${}_{B.1}^{\mathrm{CON}-3}$	${}_{A.1}^{\mathrm{SP}-1}$	${}_{A.1}^{\mathrm{CV}-1}$	${}_{A.1}^{\mathrm{ACT}-1}$	${}_{A.1}^{\mathrm{ACT}-2}$	${}_{A.1}^{\mathrm{PV}-1}$
3.1	0	0	0	1	1	0	1	1	0	1
3.2	0	1	1	1	1	1	0	1	0	1
4.1	0	1	0	0	0	0	1	1	0	1
4.2	1	1	0	0	0	1 ^1^	1	1	0	1

^1^ Taking into account the time containing the *SP* changes.

**Table 14:** Most common diagnosis for each loop and each scenario. Red color indicates incorrect values.

	${}_{A.1}^{\mathrm{CON}-1}$	${}_{B.1}^{\mathrm{CON}-1}$	${}_{A.1}^{\mathrm{CON}-2}$	${}_{A.1}^{\mathrm{CON}-3}$	${}_{B.1}^{\mathrm{CON}-3}$	${}_{A.1}^{\mathrm{SP}-1}$	${}_{A.1}^{\mathrm{CV}-1}$	${}_{A.1}^{\mathrm{ACT}-1}$	${}_{A.1}^{\mathrm{ACT}-2}$	${}_{A.1}^{\mathrm{PV}-1}$
3.1	normal	normal	normal	CON-3	CON-3	normal	CON-12	ACTCV	normal	CON-3
3.2	normal	CON-12	CON-12	CON-3	CON-3	normal	normal	ACTCV	normal	PVSP
4.1	normal	CON-12	normal	normal	normal	normal	CON-12	CON-12	normal	PVSP
4.2	CON-12	CON-12	normal	normal	normal	PVSP ^1^	CON-12	ACTCV	normal	PVSP

^1^ Taking into account the time containing the *SP* changes.

**Table 15:** Affected loops in each scenario.

	${}_{A.1}^{\mathrm{CON}-1}$	${}_{B.1}^{\mathrm{CON}-1}$	${}_{A.1}^{\mathrm{CON}-2}$	${}_{A.1}^{\mathrm{CON}-3}$	${}_{B.1}^{\mathrm{CON}-3}$	${}_{A.1}^{\mathrm{SP}-1}$	${}_{A.1}^{\mathrm{CV}-1}$	${}_{A.1}^{\mathrm{ACT}-1}$	${}_{A.1}^{\mathrm{ACT}-2}$	${}_{A.1}^{\mathrm{PV}-1}$
3.1	0	0	0	1	1	0	1	1	1	1
3.2	0	1	1	1	1	1	0	1	1	1
4.1	0	0	0	0	0	0	1	0	1	1
4.2	1	1	0	0	0	1	0	0	1	1

**Table 16:** Settings estimation for scenario ${}_{A.1}^{CON-3}$ under different disturbances and cyber-fault sizes.

		$25\%{\mathrm{cf}}^{C.S}$	$50\%{\mathrm{cf}}^{C.S}$	$150\%{\mathrm{cf}}^{C.S}$	50%dist	150%dist
3.1	$k_{p}$	−5	−10	−30	−20	−20
	${\hat{k}}_{p}$	−4.66	−8.01	−19.93	−12.88	−13.13
	$T_{i}$	152	76	25	38	38
	${\hat{T}}_{i}$	144.81	66.59	39.19	34.66	36.44
3.2	$k_{p}$	7.5	15	45	30	30
	${\hat{k}}_{p}$	7.24	15.05	48.79	31.20	31.27
	$T_{i}$	152	76	25	38	38
	${\hat{T}}_{i}$	147.46	81.54	41.18	45.28	50.23
4.1	$k_{p}$	−5	−5	−5	−5	−5
	${\hat{k}}_{p}$	−4.54	−4.50	−4.52	−4.54	−4.51
	$T_{i}$	170	170	170	170	170
	${\hat{T}}_{i}$	155.51	159.82	160.03	163.00	158.09
4.2	$k_{p}$	5	5	5	5	5
	${\hat{k}}_{p}$	4.91	4.88	4.71	4.66	4.83
	$T_{i}$	90	90	90	90	90
	${\hat{T}}_{i}$	89.12	89.32	85.44	85.01	87.75

**Table 17:** Detection and isolation for scenario ${}_{A.1}^{CON-3}$ under different disturbances and cyber-fault sizes.

Scenario		3.1	3.2	4.1	4.2
$25\%cf_{3.1}^{C.S}$, $25\%cf_{3.2}^{C.S}$	%detection	46.49	71.31	1.74	0.69
	%correct	36.38	65.39	98.26	99.31
$50\%cf_{3.1}^{C.S}$, $50\%cf_{3.2}^{C.S}$	%detection	99.31	99.31	0.0	0.0
	%correct	81.09	95.05	100.0	100.0
$150\%cf_{3.1}^{C.S}$, $150\%cf_{3.2}^{C.S}$	%detection	99.45	98.40	0.0	0.0
	%correct	36.00	42.33	100.0	100.0
50%dist	%detection	99.35	99.49	0.0	6.24
	%correct	76.38	63.29	100.0	93.76
150%dist	%detection	99.38	98.37	0.0	4.64
	%correct	47.63	68.38	100.0	95.36

## Data Availability

Data are available at https://doi.org/10.5281/zenodo.7612269.

## Abbreviations

The following abbreviations are used in this manuscript:

PV	Process Variable
SP	Set Point
CV	Control Signal
ICS	Industrial Control Systems
IDS	Intrusion Detection Systems
CPS	Cyber-Physical Systems
FDI	Fault Detection and Isolation
IAE	Integral Absolute Error
MW	Moving Window
EWMA	Exponentially Weighted Moving Average
RW	Rolling Window

## Notations

List of simulated cybernetic faults.

$cf_{3.2}^{C.M}$, $cf_{4.2}^{C.M}$	Changing the operating mode (to “manual”) of the indicated control loop
$cf_{3.2}^{CV.C}$, $cf_{4.2}^{CV.C}$	Modification (type *C*) of the control value CV of the indicated loop
$cf_{3.1}^{C.R}$, $cf_{3.2}^{C.R}$	Changing the operating mode (to “reverse”) of the indicated control loop
$cf_{3.1}^{C.S}$, $cf_{3.2}^{C.S}$	Changing the settings ($k_{p}$, $T_{i}$) of the indicated control loop
$cf_{3.2}^{SP.C}$, $cf_{4.2}^{SP.C}$	Modification (type *C*) of the setpoint SP of the indicated loop
$cf_{3.1}^{CV.P}$, $cf_{4.1}^{CV.P}$	Modification (type *P*) of the control value CV of the indicated loop
$cf_{3.1}^{PV.C}$, $cf_{3.2}^{PV.C}$, $cf_{4.1}^{PV.C}$, $cf_{4.2}^{PV.C}$	Modification (type *C*) of the process value PV of the indicated loop
$cf_{3.1}^{PV.P}$, $cf_{3.2}^{PV.P}$, $cf_{4.1}^{PV.P}$, $cf_{4.2}^{PV.P}$	Modification (type *P*) of the process value PV of the indicated loop
$cf_{V_{3}}^{A.O}$, $cf_{V_{4}}^{A.O}$	Taking control of the operation of the indicated actuator
$cf_{V_{4}}^{A.S}$	Changing the operating parameters (dead band) of the indicated actuator
